# Plasma Concentrations of Endotoxin Lipopolysaccharide and High-Mobility Group Box 1 Protein Are Consistent Sex-Specific Biomarkers of Alcohol Abstinence Associated with Alcohol Use Disorder

**DOI:** 10.3390/toxics14050440

**Published:** 2026-05-15

**Authors:** Isaac Hurtado-Guerrero, Nuria García-Marchena, Jaime Martín-Martín, María Flores-López, Nerea Requena-Ocaña, María del Mar Fernández-Arjona, Antonio J. López-Gambero, Patricia Rivera, Leticia Rubio, Gabriel Rubio, Antonia Serrano, Fernando Rodríguez de Fonseca, Juan Suarez

**Affiliations:** 1 Instituto de Investigación Biomédica de Málaga y Plataforma en Nanomedicina-IBIMA Plataforma BIONAND, 29590 Málaga, Spain; isaac.hurtado@uma.es (I.H.-G.); ngmarchena@ucm.es (N.G.-M.); jaimemartinmartin@uma.es (J.M.-M.); maria.flores@ibima.eu (M.F.-L.); nereareq@uma.es (N.R.-O.); marfernandez@uma.es (M.d.M.F.-A.); antonio.lopez@ibima.eu (A.J.L.-G.); patriciarivera@uma.es (P.R.); lorubio@uma.es (L.R.); antonia.serrano@ibima.eu (A.S.); 2 Departamento de Anatomía Humana, Medicina Legal e Historia de la Ciencia, Universidad de Málaga, 29071 Málaga, Spain; 3 Departamento de Psicobiología, Facultad de Psicología, Universidad Complutense de Madrid, Pozuelo de Alarcón, 28223 Madrid, Spain; 4 UGC Salud Mental, Hospital Regional Universitario de Málaga, 29010 Málaga, Spain; 5 Departamento de Psicobiología, Facultad de Psicología, Universidad de Málaga, 29071 Málaga, Spain; 6 Departamento de Biología Celular, Genética y Fisiologia, Universidad de Málaga, 29071 Málaga, Spain; 7 UGC Neurología, Hospital Regional Universitario de Málaga, 29010 Málaga, Spain; 8 Departamento de Psiquiatria, Hospital 12 de Octubre, Insituto i+12, 28223 Madrid, Spain; gabriel.rubio@salud.madrid.org

**Keywords:** alcohol, endotoxin, inflammation, abstinence, forensic toxicology

## Abstract

Alcohol use disorder (AUD) is associated with gut dysbiosis through interactions with the immune system. The present study aimed to investigate whether endotoxin lipopolysaccharides (LPS) and high-mobility group box-1 protein (HMGB1), a key inflammatory mediator, as well as the metabolic fat mass hormone leptin, are reliable biomarkers for the estimation of alcohol dependence and abstinence. AUD outpatients (N = 122) and healthy volunteers (N = 63) were recruited and assessed by using the Psychiatric Research Interview for Substance and Mental Disorders according to DSM-IV-TR after blood extraction. The results indicated that AUD patients had higher plasma concentrations of LPS and HMGB1, and lower plasma concentrations of leptin and SDF-1α compared to healthy subjects. Two logistic models, including HMGB1, leptin and SDF-1α (model 1) or LPS (model 2), provided high discriminatory powers to identify AUD patients [prognostic probability: model 1 = 0.90 (0.78); model 2 = 0.86 (0.79); *p* < 0.001]. LPS and HMGB1 positively correlated with alcohol abstinence duration in male AUD patients only. Linear logistic regression included LPS, HMGB1, fractalkine, SDF-1α and/or leptin to accurately estimate the duration of problematic alcohol use and alcohol abstinence when sexes were analyzed separately. These results suggest that LPS and HMGB1 are relevant sex-specific actors for predicting alcohol abstinence and problematic alcohol use in AUD patients.

## 1. Introduction

At-risk alcohol use is a major contributor to disease, injury and mortality worldwide, with particularly high prevalence in the European Union, where it imposes a substantial social, healthcare and economic burden [1,2]. According to the Global Burden of Disease 2019 study, alcohol consumption was identified as the leading risk factor for disability-adjusted life years among individuals aged 25–49 years worldwide [3]. Projections suggest that alcohol-related mortality will stagnate or even increase among middle-aged and older adults by 2050 [4]. Among the diverse pathophysiological effects of alcohol—encompassing neuropsychiatric, hepatic, cardiovascular, and malignant conditions—gastrointestinal (GI) diseases represent a major contributor to alcohol-associated mortality [5].

Several mechanisms underlie alcohol-induced GI dysfunction [5]. Chronic alcohol misuse was shown to impair hepatic bile production, alter bile acid physiology, and interfere with bacterial biotransformation processes, collectively contributing to liver injury [6]. Recent evidence also indicates that alcohol consumption directly compromises intestinal barrier integrity and alters both the composition and function of the gut microbiome, primarily through heightened immune activation [7,8,9,10,11]. Alcohol promotes gut bacterial overgrowth and reduces both α- and β-diversity, accompanied by a reduction in beneficial *Lactobacilli* species. These alterations lead to dysbiosis and increased intestinal permeability, facilitating the translocation of bacteria and endotoxins into the systemic circulation. Additionally, alcohol-induced gut permeability is exacerbated by the disruption of epithelial cell junctions, likely driven by enhanced bacterial metabolism of alcohol and elevated acetaldehyde concentrations in the intestinal lumen [5]. Notably, fecal microbiota transplantation has been employed to mitigate endotoxemia and restore eubiosis and intestinal barrier integrity in both alcohol-exposed animal models and clinical trials targeting liver diseases [12,13,14].

Recent evidence indicates that alcohol-induced dysbiosis and the resulting production of bioactive compounds contribute to exacerbated immune responses that adversely affect central nervous system functions, leading to cognitive deficits, mood disturbances, and depression [15,16,17,18]. Because males and females exhibit distinct innate and adaptive immune profiles—and given that most research on alcohol use disorder (AUD) has predominantly focused on male populations [19,20]—there is increasing recognition of sex as a critical biological variable in AUD research. Notably, female patients with AUD are at a greater risk than males for developing metabolic disorders, cancer, cardiovascular issues, and gastrointestinal disease [21]. In this context, evidence suggests that leptin interacts with dopaminergic signaling, which is critically involved in substance use behaviors [22,23,24]. In the context of AUD, leptin levels are more altered in females than in males, and these sex-specific changes may contribute differently to craving, reward sensitivity, stress reactivity and neuroimmune dysregulation, factors that are central to the development and maintenance of alcohol dependence [25,26,27,28].

Although alcohol withdrawal is known to disrupt gut microbial integrity, the specific contribution of endotoxin components in the context of alcohol dependence, abstinence, and potential sex-related differences remains poorly defined. In addition, although preliminary studies suggest that sex differences in biomarkers of tissue damage associate binge drinking with immune, metabolic and endocrine function, their limited sample sizes preclude definitive conclusions [29]. In this exploratory study, we quantified plasma concentrations of lipopolysaccharide (LPS) and inflammatory mediators, including cytokines and chemokines released by immune cells, as potential biomarkers for assessing the duration of problematic alcohol use and abstinence in male and female AUD patients.

## 2. Materials and Methods

### 2.1. Ethics Statements

All participants provided written informed consent after receiving a detailed description of the study and were allowed to ask questions and express any concerns. The study protocol and recruitment procedures were approved by the Regional Ethics Committee (PEIBA, reference no. PND2019/040, approval date: 14 April 2021), in accordance with the ethical principles outlined in the Declaration of Helsinki (64th WMA General Assembly, Fortaleza, Brazil, 2013) and the General Data Protection Regulation 2016/679 of the European Union. To ensure confidentiality and privacy, all data were anonymized and assigned coded identifiers.

### 2.2. Participants and Recruitment

This cross-sectional study included 185 Caucasian participants divided into two groups: 63 healthy control subjects (control group) and 122 outpatients diagnosed with alcohol use disorder (AUD group). The AUD group consisted of individuals enrolled in outpatient treatment programs at Hospital Universitario 12 de Octubre (Madrid, Spain) and Hospital Regional Universitario de Málaga (Málaga, Spain), forming part of a cohort within the Spanish Network for Addictive Disorders (Red de Trastornos Adictivos, RETICS, Instituto de Salud Carlos III). The AUD group was further divided into 97 male and 25 female patients. Control subjects were recruited from a cohort of volunteers employed at the Spanish National Public Health System (Hospital Regional Universitario de Málaga, Málaga, Spain). They were matched to the AUD group by body mass index (BMI) and sex.

### 2.3. Eligibility Criteria

Participation was voluntary, and all participants met the following inclusion criteria: age between 18 and 65 years, and, for the AUD group, a lifetime diagnosis of alcohol use disorder with a minimum of 4 weeks of abstinence. Despite no biochemical alcohol or related metabolites being obtained, abstinence ≥4 weeks was ensured through structured clinical interviews at screening and baseline, corroborated by collateral information when available. Exclusion criteria included a medical history of chronic inflammatory disorders (e.g., cancer, coronary artery diseases, atherosclerosis, diabetes, arthritis, asthma, chronic obstructive pulmonary disease, neurodegenerative diseases, or gastrointestinal and hepatobiliary diseases), infectious diseases (including HIV, hepatitis B, hepatitis C, or COVID-19), cognitive or language impairments precluding evaluation, pregnancy or breastfeeding, and current substance use other than nicotine or caffeine. For the control group, additional exclusion criteria were a personal history of psychiatric disorders or problematic use of addictive substances.

### 2.4. Clinical Evaluations

All participants were assessed through structured psychiatric interviews conducted by experienced clinical psychologists, using an instrument appropriate to each sample group. For outpatients, the Spanish version of the “Psychiatric Research Interview for Substance and Mental Diseases” (PRISM) was administered. PRISM is a semi-structured interview based on the “Diagnostic and Statistical Manual of Mental Disorders, Fourth Edition, Text Revision” (DSM-IV-TR) criteria, with strong psychometric properties for diagnosing substance-induced and major mental disorders in individuals with addictions, demonstrating good to excellent validity and test–retest reliability [30,31,32,33]. Data collected included variables related to abstinence duration, severity of substance use disorder (SUD)—calculated by summing DSM-IV-TR diagnostic criteria—mental and medical comorbidities, and use of psychotropic medication. Control participants were evaluated using the Spanish version of the Composite International Diagnostic Interview (CIDI) to identify mental disorders [34], along with the PRISM module 1: Overview for sociodemographic and physiological variables [35].

### 2.5. Collection and Processing of Plasma Samples

Blood samples were collected in the morning by trained nurses after overnight fasting and prior to clinical evaluations. Venous blood samples were drawn into 10 mL K2 EDTA tubes (BD, Franklin Lakes, NJ, USA) and centrifuged at 2200× *g* for 15 min at 4 °C to obtain plasma. Each plasma samples were screened for infectious diseases using commercial assays for HIV, hepatitis B, hepatitis C (Strasbourg, Cedex, France), and SARS-CoV-2 (Bio-Connect, Huissen, The Netherlands). In accordance with laboratory safety standards, samples testing positive were discarded. Plasma was stored at −80°C until analysis.

### 2.6. Determination of Endotoxin Lipopolysaccharide (LPS)

Plasma concentrations of endotoxin lipopolysaccharide (LPS) were quantified in duplicate using the Thermo Scientific™ Pierce™ Chromogenic Endotoxin Quant Kit (cat. no. A39553; Rockford, IL, USA) according to the manufacturer’s instructions. This assay is a quantitative endpoint test designed for the detection of LPS, a membrane component of Gram-negative bacteria, utilizing amebocyte lysates derived from horseshoe crab blood. Upon exposure to endotoxin, a cascade of enzymatic reactions activates Factor C, Factor B and pro-clotting enzyme. The activated enzyme then catalyzes the release of p-nitroaniline (pNA) from the colorless chromogenic substrate Ac-Ile-Glu-Ala-Arg-pNA, resulting in a yellow product. After stopping the reaction, the released pNA was measured photometrically at 405 nm. The assay shows a linear correlation between absorbance and endotoxin concentration in the ranges of 0.01–0.1 EU/mL and 0.1–1.0 EU/mL (prior to sample dilution; a 1:50 dilution was recommended). The color intensity is directly proportional to the endotoxin concentration, as determined from a standard curve with a coefficient of determination (R^2^) of 0.96. The intra-assay coefficient of variation (CV) was <3%.

### 2.7. Determination of Inflammatory Factors

Plasma concentrations of HMGB1 (high-mobility group protein B1) were quantified using a colorimetric human HMGB1/HMG-1 sandwich ELISA kit (cat. no. NBP2-62766; Novusbio, Abingdon, UK). The assay employs a biotinylated detection antibody specific to human HMGB-1 and an avidin–horseradish peroxidase (HRP) conjugate. After the addition of the substrate reagent and stop solution, optical density (OD) was measured at 450 nm ± 2 nm. The kit reports a sensitivity of 18.75 pg/mL and a detection range of 31.25–2000 pg/mL, excluding the sample dilution factor (recommended 1:50). The intra-assay and inter-assay CV were <5.09% and <4.52%, respectively.

### 2.8. Determination of Leptin

Plasma leptin concentrations were quantified using the Human Leptin Quantikine^TM^ ELISA kit (cat. no. DLP00; R&D Systems, Abingdon, UK), according to the manufacturer’s instructions. The assay employs a monoclonal antibody specific to human leptin conjugated to HRP. Following the addition of substrate solution and stop solution, the OD was measured at 450 nm. Concentrations were calculated considering a recommended 1:100 dilution. The kit reports a sensitivity of 7.8 pg/mL and a detection range of 15.6–1000 pg/mL. The intra-assay and inter-assay CV were <3.3% and <5.4%, respectively.

### 2.9. Multiplexed Bead Immunoassay

Plasma concentrations of fractalkine, MCP-1, SDF-1α, IL-1β, IL-6, IL-10 and TNFα were measured using Human High Sensitivity ProcartaPlex^TM^ Multiplex Cytokine and Chemokine Panels (cat. no. PPX-07-MXH6ANW and cat. no. EPXS090-12199-901, Invitrogen, Waltham, MA, USA) in a Luminex xMAP^®^ technology—MAGPIG system (ThermoFisher, Waltham, MA, USA). Sensitivity was approximately 39, 51, 13, 18, 25, 28 and 45 pg/mL for fractalkine, MCP-1, SDF-1α, IL-1β, IL-6, IL-10 and TNF-α, respectively. Mean intra-assay variation (%CV replicates) was 36.8, 25.3, 3.8, 19.5, 13.8 and 26.2%, respectively. Minimum detectable concentration values were attributed to missing values that were below the standard curve.

### 2.10. Biochemical Analysis

The plasma concentrations of the hepatic enzymes gamma-glutamyltransferase (GGT), glutamic oxaloacetic transaminase (GOT) and glutamate pyruvate transaminase (GPT) were measured using commercial kits according to the manufacturer’s instructions (cat. no. DF45A, DF41A and DF43A, respectively, Flex^®^ reagent cartridge, Dimension, Siemens Healthcare GmbH, Erlangen, Germany) according to the manufacturer’s instructions in a Siemens Dimension Vista 500 Lab System (Siemens Healthcare GmbH). In all cases, a calibration curve and internal controls were included in each assay. Liver enzyme levels were expressed in IU/L.

### 2.11. Statistical Analysis

Data in tables were expressed as the number and percentage of subjects [N (%)] and mean and standard deviations (mean ± SD). The Shapiro–Wilk test indicated that data from continues variables were not normally distributed. The significance of differences was determined using the chi-square test for categorical variables and the Mann–Whitney U test for continuous variables. Spearman’s rho for correlation analysis between plasma concentration of inflammatory biomarkers and relevant AUD-related variables (age at first alcohol use, alcohol abstinence and problematic alcohol use) was performed. Kolmogorov–Smirnov statistics were used to determine cutoff points. A receiver operating characteristic (ROC) analysis for discriminative binary logistic regression models and adjusted for sex and age as covariates was performed to identify alcohol-abstinent patients of both sexes. Backward elimination was performed within a bootstrap framework of 5000 successful resamples to identify stable predictor combinations. The resulting prognostic variability of each model was analyzed using the Mann–Whitney U test. Finally, we performed multiple linear regression analyses with a backward stepwise entry method to determine biomarker predictors of problematic alcohol use and alcohol abstinence. *p*-values less than 0.05 were considered statistically significant. Statistical analyses were carried out using JASP version 0.19 software.

## 3. Results

### 3.1. Sociodemographic and Clinical Characteristics of AUD and Control Groups

Both the control and AUD cohorts showed similar body mass index (BMI), although the AUD group was predominantly composed of older subjects compared with the control group (*p* < 0.001). The AUD group is formed by a similar percentage of female participants than the control group, but differences were observed in marital status (*p* < 0.001), education degree (*p* < 0.001) and occupation (*p* < 0.001) (Table 1).

Among the clinical characteristics of patients with AUD (Table 2), tumors were the most prevalent medical problem (43.1%), especially in females (64%), followed by circulatory or cardiac (17.1%) and digestive problems (24.4%). In total, 66.4% of the AUD patients (60.8% of males and 88% of females) had comorbid psychiatric disorders such as mood, anxiety, attention deficit hyperactivity disorder (ADHD), borderline personality (BP), psychotic disorder or antisocial personality (AP). Almost all the AUD patients received lifespan psychiatric or psychological treatment, and 77.2% of them took psychiatric medication, more frequently females.

Among the psychiatric disorders diagnosed in AUD patients (Table 3), mood disorders were the most prevalent (43.3%), followed by anxiety (22.3%), ADHD (17.3%), BP (14.1%), psychotic disorders (7.5%) and antisocial personality (4.1%). Mood disorders were significantly more frequent in females than in males (*p* < 0.01).

The variables characterizing addiction according to the DSM-IV-TR and PRISM for AUD patients (Table 4) indicated that the mean age of alcohol use onset was 30.51 years. In comparison, the average duration of problematic alcohol use was 16.38 years. Male patients reported a significantly lower mean age of alcohol use onset compared with female patients (*p* < 0.05). The mean duration of alcohol abstinence among AUD patients was 10 months. The mean score of alcohol abuse, according to diagnostic criteria, was 7.34 in the AUD group and was significantly lower in females compared with males (*p* < 0.05). Among AUD patients, 76% suffered no or one period of abstinence. In total, 92.6% of AUD patients were diagnosed with alcohol abuse and dependency throughout their life, 91.8% in the last 12 months, and 52.5% in the last year. In addition to alcohol, 14.8% of AUD patients also reported abuse and dependency on cocaine (16.5% of males and 8% of females, *p* < 0.05), and 14.8% reported use of cannabis, 6.5% sedatives, and 3.3% use of stimulants (Table 4).

### 3.2. Plasma Concentrations of Inflammation-Related Factors in Abstinent AUD and Control Subjects

We analyzed a panel of plasma inflammatory mediators including cytokines and chemokines from immune cells to assess the duration of problematic alcohol use, including lipopolysaccharide (LPS), high-mobility group box 1 (HMGB1), leptin, fractalkine, monocyte chemoattractant protein 1 (MCP-1), stromal cell-derived factor 1 alpha (SDF-1α), interleukin-1 beta (IL-1β), -6 (IL-6), -10 (IL-10), and tumor necrosis factor alpha (TNF-α). We compared the results obtained in the AUD and control groups (Table 5). Statistical analysis revealed that AUD patients had higher plasma concentrations of LPS (*U* = 3289; *p* = 0.013), HMGB1 (*U* = 4987; *p* ˂ 0.001) and IL-6 (*U* = 217; *p* = 0.012), and lower plasma concentrations of leptin (*U* = 1922; *p* = 0.003) compared with controls (Table 5). The liver enzymes GGT, GOT and GPT were also analyzed, as they are routinely used clinical markers of alcohol-related liver injury. Plasma GGT levels differed significantly between AUD individuals and controls (*U* = 433.5; *p* < 0.001), whereas GOT and GPT showed no significant group differences. Importantly, all enzyme values remained within their respective reference ranges, indicating the absence of overt hepatic pathology in either group.

When analyses were stratified by sex, statistics revealed higher plasma concentrations of HMGB1 (*U* = 1098; *p* < 0.001), IL-1β (*U* = 49; *p* = 0.045), IL-6 (*U* = 54; *p* = 0.007) and TNF-α (*U* = 49; *p* = 0.045) in male AUD patients compared with male controls (Table 6).

In contrast, our findings revealed that the female AUD group exhibited higher concentrations of LPS (*U* = 567; *p* = 0.012) and HMGB1 (*U* = 577; *p* = 0.017) compared with the female control group (Table 7). No further significant differences were observed in the other factors analyzed.

### 3.3. Inflammation-Related Factors as Predictors of Abstinent AUD

To differentiate patients diagnosed with AUD from healthy control subjects, a logistic regression analysis was conducted utilizing the plasma concentrations of inflammation-related factors (Table 8).

**Figure 1:**
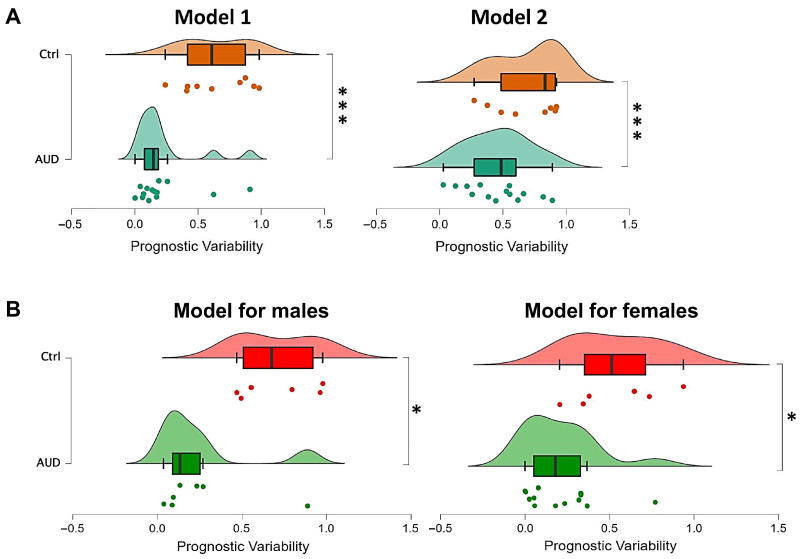
Scatter plots for prognostic variability of final models of AUD. (**A**) Predicted probabilities of Model 1 (HMGB1, leptin and SDF-1α) and Model 2 (HMGB1, leptin and LPS), after ROC analysis, that differentiate AUD patients from controls. (**B**) Predicted probabilities of a Model for male (HMGB1 and TNF-α) and a Model for female (LPS and fractalkine), after ROC analysis that differentiates male AUD patients from male controls, as well as female AUD patients from female controls, respectively. Mann–Whitney U-test: (*) *p* < 0.05 and (***) *p* < 0.001, compared with the control group.

The receiver operating characteristic (ROC) analysis identified two highly effective discriminative models distinguishing AUD patients from control: Model 1, comprising the HMGB1, leptin and SDF-1α, achieved a prognostic probability of 78% with an area under the curve (AUC) of 0.904 (95% confidence interval (CI): 0.867–0.889). Model 2, which included HMGB1, leptin and LPS, yielded a prognostic probability of 79% and an AUC of 0.861 (95% CI: 0.841–0.711). The predicted probabilities generated of both models further demonstrated significant disparities between AUD patients and controls (*p* < 0.001; Figure 1A). Importantly, the inclusion of GGT in the ROC analysis further enhanced the discriminative performance of both models. (Table 8). An analogous analysis was executed for male and female subjects separately to distinguish male AUD patients from male controls, as well as female AUD patients from female controls. The model for male subjects included the variables HMGB1 and TNF-α (prognostic probability = 72%, AUC = 0.905, CI = 1–0.857), whereas the model for female subjects included the variables LPS and fractalkine (prognostic probability = 68%, AUC = 0.859, CI = 0.833–0.846). The predicted probabilities for both male and female models also highlighted significant differences between male AUD patients and male controls, as well as between female AUD patients and female controls (*p* < 0.05; Figure 1B).

### 3.4. Correlations Between the AUD Defining Variables and Plasma Concentrations of Inflammation-Related Factors

**Figure 2:**
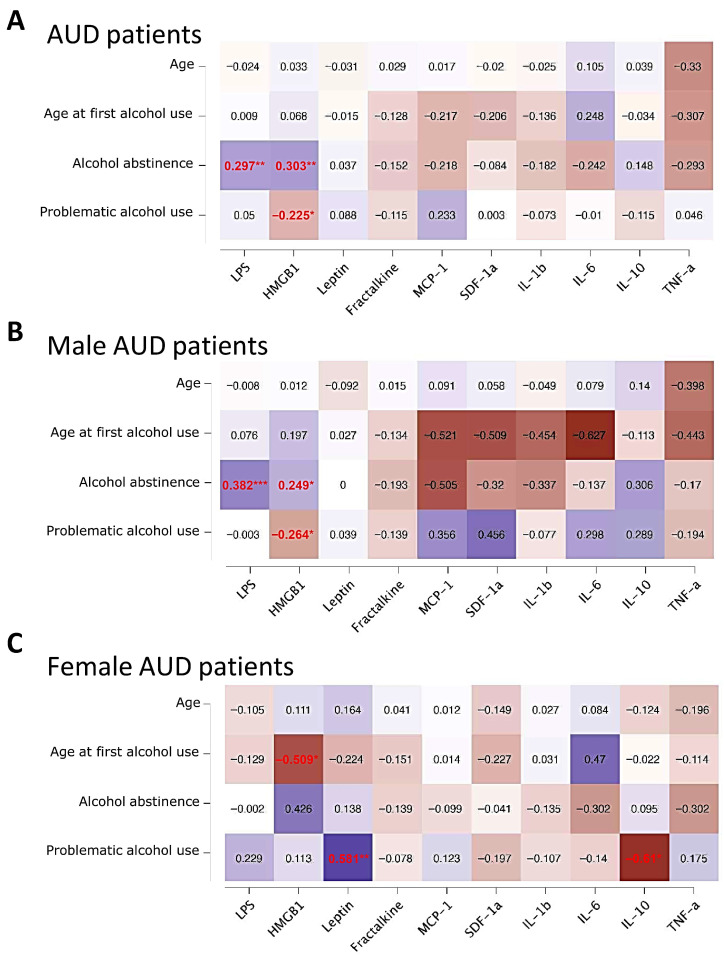
Heat maps showing correlation analysis between selected AUD-related variables (age, age at first alcohol use, alcohol abstinence and problematic alcohol use) and the plasma concentration of inflammatory biomarkers (LPS, HMGB1, leptin, fractalkine, MCP-1, SDF-1α, IL-1β, IL-6, IL-10 and TNF-α) in AUD patients (**A**), and AUD patients segregated by sex (**B**,**C**). The colors, ranging from blue to brown, show the positive and negative Spearman’s rho correlation coefficients. Coefficients in red show significant correlations: (*/**/***) *p* < 0.05/0.01/0.001.

To assess the potential association between variables characterizing AUD and plasma concentrations of inflammation-related biomarkers, we conducted a correlation analysis. The correlation coefficients were visualized using heat maps (Figure 2). This analysis disclosed a significant positive correlation between plasma concentrations of LPS and HMGB1 and the duration of alcohol abstinence (R = 0.297 and 0.303, respectively; *p* < 0.01; Figure 2A). Furthermore, the plasma concentrations of HMGB1 were negatively correlated with problematic use of alcohol (R = −0.225, *p* < 0.05; Figure 2A). No significant correlation was found between hepatic enzymes (GGT, GOT and GPT) and addiction-related parameters. Sex-stratified analyses yielded distinct patterns. In the male AUD group, similar positive correlations were observed between plasma concentrations of LPS and HMGB1 and alcohol abstinence (R = 0.382, *p* < 0.001; R = 0.249, *p* < 0.05, respectively; Figure 2B). Additionally, HMGB1 concentrations exhibited a negative correlation with problematic use of alcohol (R = −0.264, *p* < 0.05; Figure 2B). In contrast, among AUD females, plasma leptin concentrations were positively correlated with problematic alcohol use (R = 0.581, *p* < 0.01), while HMGB1 concentrations were negatively correlated with the age at first alcohol use (R = −0.509, *p* < 0.05). Furthermore, IL-10 concentrations were inversely correlated with problematic alcohol use (R = −0.61, *p* < 0.05; Figure 2B).

To assess the predictive value of inflammatory markers for AUD-related behavioral variables, we performed multiple linear regression analyses with a backward stepwise entry method. This model identified HMGB1, fractalkine, MCP-1, SDF-1α and TNF-α as significant predictors of problematic alcohol use in the overall AUD cohort (*F*_5,8_ = 7.586, *p* = 0.007), explaining 71.1% of the variance:

Problematic alcohol use = −22.204 + (0.468 × HMGB1) + (1.799 × Fractalkine) + (−0.327 × MCP-1) + (−0.074 × SDF-1α) + (1.465 × TNF-α)

Using the same statistical approach, alcohol abstinence was predicted by LPS, HMGB1, MCP-1 and IL-6 (*F*_4,9_ = 3.507, *p* = 0.05), accounting for 43.6% of the variance:

Alcohol abstinence = −7.971 + (−22.326 × LPS) + (1.098 × HMGB1) + (−1.153 × MCP-1) + (−1.391 ×IL-6)

When analyses were stratified by sex, distinct predictive patterns emerged. In male AUD patients, HMGB1 alone significantly predicted problematic alcohol use (*F*_1,662_ = 5.285, *p* = 0.025), explaining 88.0% of the variance:

Male problematic alcohol use = −19.488 + (−6.241 × 10^−4^ × HMGB1)

Similarly, LPS and HMGB1 together predicted alcohol abstinence in males (*F*_2,63_ = 8.507, *p* < 0.001), explaining 95.1% of the variance:

Male alcohol abstinence = −4.312 + (0.011 × LPS) + (0.001 × HMGB1)

In female AUD patients, problematic alcohol use was best predicted by LPS, HMGB1, fractalkine and SDF-1α (*F*_4,4_ = 13.297, *p* = 0.014), accounting for 99.3% of the variance:

Female problematic alcohol use = −6.024 + (0.004 × LPS) + (9.851 × 10^−4^ × HMGB1) + (0.012 × fractalkine) + (2.992 × 10^−4^ × SDF-1α)

Finally, alcohol abstinence in females was significantly predicted by leptin and LPS concentrations (*F*_2,6_ = 7.625, *p* = 0.023), explaining 93.8% of the variance:

Female alcohol abstinence = −48.152 + (0.019 × leptin) + (−0.024 × LPS)

## 4. Discussion

Current diagnostic approaches for AUD primarily rely on self-assessment and clinical interviews, which are inherently subjective and susceptible to bias. In this context, the identification of objective, biologically grounded biomarkers offer a promising avenue to enhance diagnostic precision, guide personized treatment strategies, and facilitate longitudinal monitoring of disease progression and recovery. In this exploratory study, we investigated peripheral blood concentrations of LPS (a gut-derived endotoxin indicative of microbial translocation and dysbiosis), alongside a panel of selected immune mediators, including cytokines and chemokines, in male and female individuals diagnosed with AUD. Our aim was to evaluate the potential of these biomarkers to predict the duration of problematic alcohol use and abstinence, thereby contributing to the development of biologically informed tools for clinical assessment and stratification in AUD.

The main findings indicate that AUD patients exhibited elevated plasma concentrations of LPS, HMGB1 and IL-6, alongside reduced leptin levels, when compared to healthy controls. Sex-stratified analyses revealed that male AUD patients had significantly higher plasma concentrations of HMGB1, IL-1β, IL-6 and TNF-α relative to male controls. In contrast, female AUD patients showed elevated concentrations of LPS and HMGB1. Based on these biomarkers, two predictive models were developed to discriminate AUD. The first model, comprising HMGB1, leptin, and SDF-1α, achieved a prognostic accuracy of 78%. The second model, which included HMGB1, leptin and LPS, yielded a prognostic probability of 79%. Notably, the discriminative performance of both models improved further when GGT was incorporated into the respective ROC analysis (prognostic probability of 90 and 91%, respectively). Sex-specific models further refined predictive capacity: in males, a model incorporating HMGB1 and TNF-α reached a 72% accuracy, while in females, a model consisting of LPS and fractalkine achieved 68% accuracy in distinguishing AUD patients from controls. Notably, elevated plasma concentrations of LPS and HMGB1 were positively correlated with more extended periods of alcohol abstinence, particularly among male AUD patients. Conversely, female AUD patients with higher leptin and lower IL-10 levels exhibited more severe alcohol-related problems. Furthermore, increased HMGB1 concentrations were significantly correlated with an earlier age of alcohol initiation in female patients.

The present study indicates that LPS, HMGB1, IL-6, and leptin are key mediators of the inflammatory processes underlying AUD, leading to organ damage perpetuating alcohol dependence. Chronic alcohol consumption compromises gut barrier integrity, facilitating translocation of LPS (an endotoxin derived from the outer membrane of Gram-negative bacteria) into the bloodstream [36,37]. Circulating LPS activates innate immune cells, triggering the release of pro-inflammatory mediators such as HMGB1, which in turn amplify systemic inflammation and contribute to pathological changes in the liver, brain, and pancreas [5,38]. HMGB1 plays a pivotal role in alcohol-induced neuroinflammation and has been implicated in both the initiation and maintenance of alcohol dependence. Elevated serum HMGB1 levels correlated positively with AUD severity, including alcohol-associated liver disease, and may serve as a clinically relevant biomarker [39]. Additionally, IL-6, a pro-inflammation cytokine secreted by macrophages in response to gut-derived microbial products such as LPS, is found at increased concentrations in abstinent AUD patients. This suggests a sustained neuroimmune activation during early recovery and supports a potential role for IL-6 in AUD-derived pathologies [40,41,42].

The correlation analysis examining the relationship between AUD-defining variables and inflammation-related biomarkers revealed a positive correlation between plasma LPS and HMGB1 concentrations with the duration of alcohol abstinence, particularly in male AUD patients. These results suggest that both LPS and HMGB1 are reduced during active drinking and gradually increase with sustained abstinence, reflecting dynamic immune recovery [43]. Furthermore, a negative correlation between plasma HMGB1 levels and problematic alcohol use further substantiates immune suppression during active or heavy drinking [43]. These results suggest that HMGB1 may be more useful as a biomarker of disease trajectory that a direct mediator of drinking behavior. These associations were consistent across the full AUD cohort and within the male subgroup. Notably, sex-specific analysis in female AUD patients revealed a positive correlation between leptin concentrations and problematic alcohol use, suggesting a potential role for this adipokine in modulating alcohol-related behaviors or craving in females. Additionally, a negative correlation between HMGB1 levels and age at first alcohol use in female AUD patients implies that earlier initiation of alcohol use may predispose to elevated HMGB1 levels and increased inflammatory burden. Collectively, these findings underscore distinct sex-dependent patterns in the relationship between inflammatory biomarkers and clinical features of AUD.

While LPS and HMGB1 demonstrated consistent associations with alcohol abstinence and problematic use of alcohol across sexes, leptin (a hormone secreted by adipose tissue that regulates energy homeostasis and appetite) may contribute uniquely to the alcohol abstinence in female patients with AUD. The present study indicated that leptin is reduced in abstinent AUD patients, with lower leptin levels being associated with heightened alcohol craving and consumption [23,44]. Beyond its established role in metabolic regulation and obesity, emerging evidence suggests that leptin modulates AUD-related processes through its interactions with mesolimbic reward circuitry, neuroendocrine signaling, and stress-responsive pathways. Specifically, leptin influences dopaminergic transmission in the nucleus accumbens, potentially affecting reinforcing properties of alcohol and craving [25]. Additionally, leptin participates in the regulation of the hypothalamic–pituitary–adrenal (HPA) axis, which is frequently dysregulated in individuals with AUD and may contribute to stress-induced drinking behavior [27].

Sex-specific physiological and hormonal differences, such as variations in fat distribution and estrogen regulation, may underlie the distinct leptin dynamics observed in female versus male AUD patients [25]. Notably, previous research has shown that leptin concentrations were significantly elevated in abstinent women compared to non-abstinent counterparts, particularly among those with higher BMI and longer abstinence duration [25,45]. In contrast, early-abstinent alcohol-dependent male inpatients showed lower leptin indices relative to healthy controls [46]. Consistent with these findings, our results confirmed reduced leptin concentrations in AUD patients overall and identified leptin as a positive predictor of alcohol abstinence specifically in females, supporting the hypothesis that leptin may serve as a sex-sensitive biomarker in AUD.

It is essential to note that these findings represent correlations and do not establish causality. In addition, although several confounding factors were controlled for in this study, these may still influence the observed relationships. Further research is warranted to elucidate the underlying mechanisms and to explore potential clinical implications of these results.

## 5. Conclusions

This exploratory study underscores the potential utility of LPS and HMGB1 as biologically relevant biomarkers associated with problematic alcohol use and alcohol abstinence in patients with AUD. These biomarkers reflect key pathways linking gut permeability, innate immune activation, and metabolic regulation to the clinical expression of AUD. Moreover, sex-specific differences in the plasma concentrations of inflammation-related mediators, including leptin, among patients with AUD highlight the relevance of incorporating biological sex as a critical variable in the development of diagnostic tools and personalized therapeutic strategies for addictive disorders.

Collectively, these results suggest that LPS, HMGB1 and leptin all contribute to the inflammatory processes associated with AUD and may serve as clinically informative biomarkers for disease severity, abstinence monitoring, and treatment response. However, given the cross-sectional design, sample characteristics and risk of statistical overfitting, these results should be considered preliminary and interpreted as hypothesis-generating.

## 6. Limitations

This study has several limitations that should be considered when interpreting the findings. First, the AUD cohort exhibited substantial clinical heterogeneity, including a high prevalence of psychiatric comorbidities, medical conditions and widespread use of psychotropic medication. Despite the application of strict exclusion criteria to remove major inflammatory, infectious, or hepatogastrointestinal conditions, residual co-occurring factors may influence immune activation, gut permeability, and metabolic signaling, thereby introducing additional complexity into the interpretation of between-group differences and predictive analysis. This reflects the clinical reality of AUD populations, where multimorbid conditions are highly prevalent, and often intertwined with the course and severity of the disorder. To address this, we conducted sensitivity analyses and included relevant covariates where possible to mitigate confounding effects. Additionally, the extended abstinence period (average of 10 months) was intended to reduce the acute effects of alcohol use and stabilize physiological measures, although we recognize that long-term abstinence does not eliminate all residual or comorbidity-related influences. In summary, the inflammatory signatures identified likely reflect the combined effects of AUD and its common comorbidities, and therefore cannot be interpreted as biomarkers uniquely attributable to AUD. In addition, future studies should incorporate a comprehensive panel of traditional alcohol-related biomarkers such as mean corpuscular volume (MCV) and carbohydrate-deficient transferrin (CDT) to more fully evaluate the added value.

Second, the relatively small number of female participants limits the statistical power of sex-stratified analysis. Accordingly, sex-specific associations, particularly those involving LPS and HMGB1, should be interpreted as exploratory, aimed at identifying potential trends, and require replication in larger, sex-balance cohorts. While underpowered for definitive conclusions, these preliminary observations are valuable in highlighting the need for more balanced recruitment in future studies and contribute to the broader understanding of sex-related variability in AUD-related biomarkers.

Third, from a statistical perspective, the multivariable regression models, particularly those conducted within sex-segregated subgroups, may be susceptible to overfitting due to the ratio of predictors to sample size. The absence of external validation (e.g., independent cohort testing) further restricts the predictive capacity of these models, which should be considered preliminary and interpreted as hypothesis-generating rather than definitive.

This cross-sectional design precludes causal interference, and longitudinal studies are needed to determine whether the identified biomarkers track dynamic changes in alcohol use, abstinence, and relapse risk.

## Figures and Tables

**Table 1:** Sociodemographic characteristics of the sample.

Total Sample *N* = 185				
Variables		Control Group *N* = 63	AUD Group *N* = 122	*p*-Value
Age *(Mean ± SD)*	Years	36.12 ± 13.18	50.77 ± 7.62	**˂0.001** ^a^
BMI *(Mean ± SD)*	Kg/m^2^	23.70 ± 4.17	26.26 ± 4.23	0.672 ^a^
Sex *[N (%)]*	Men	45 (65.2)	95 (79.2)	0.781 ^b^
	Women	18 (26.1)	25 (20.8)	
Marital status ^c^ *[N (%)]*	Single	30 (43.5)	30 (25)	**˂0.001** ^b^
	Cohabiting	24 (34.8)	45 (37.5)	
	Separated	5 (7.2)	41 (34.2)	
	Widow	-	4 (3.3)	
Education degree ^c^ *[N (%)]*	Unschooled	-	5 (4.2)	**˂0.001** ^b^
	Elementary	-	44 (36.7)	
	Secondary	24 (34.8)	55 (45)	
	University	35 (50.7)	16 (13.3)	
Occupation ^c^ *[N (%)]*	Employed	48 (69.6)	29 (24.2)	**˂0.001** ^b^
	Unemployed	-	20 (16.7)	
	Disability	8 (11.6)	56 (45.8)	
	Housework	-	15 (12.5)	

(^a^) *p*-value from Mann–Whitney U test. (^b^) *p*-value from chi-square test. Values in bold are statistically significant when *p* < 0.05. (^c^) Data were missing in the marital status, education degree and occupation variables. BMI, Body Mass Index.

**Table 2:** Clinical characteristics of the AUD group.

Variables *[N (%)]*		AUD *N* = 122	Male *N* = 97	Female *N* = 25	*p*-Value
Medical problems	*No*	8 (6.5)	7 (7.1)	1 (4)	**0.041** ^a^
	*Tumors*	53 (43.1)	37 (37.8)	16 (64)	
	*Endocrine/autoimmune*	7 (5.7)	4 (4.1)	3 (12)	
	*Circulatory/Cardiac*	21 (17.1)	18 (18.4)	3 (12)	
	*Digestive*	30 (24.4)	29 (29.6)	1 (4)	
	*Others*	4 (3.3)	3 (3.1)	1 (4)	
Comorbid psychiatric disorders	*No*	41 (33.6)	38 (39.2)	3 (12)	0.092 ^a^
	*Mood*	26 (21.3)	18 (18.6)	8 (32)	
	*Mood & Anxiety*	12 (9.8)	9 (9.3)	3 (12)	
	*Mood & ADHD*	4 (3.3)	4 (4.1)	0	
	*Mood & BP*	4 (3.3)	2 (2.1)	2 (8.8)	
	*Mood & Psychotic or AP*	3 (2.5)	1(1.0)	2 (8.0)	
	*Mood & ADHD & Others*	5 (4.1)	3 (3.1)	2(8.0)	
	*Anxiety*	6 (4.9)	6 (6.2)	0	
	*Anxiety & AP*	3 (2.5)	2 (2.1)	1 (4.0)	
	*Anxiety & ADHD & Others*	4 (3.1)	4 (4.1)	0	
	*ADHD*	6 (4.9)	5(5.2)	1 (4.0)	
	*ADHD & AP or Psychotic*	3 (2.5)	2 (2.1)	1 (4.0)	
	*BP or BP & AP & Psychotic*	5 (4.1)	3 (3.1)	2(8.0)	
Lifespan psychiatric or psychological treatment	*No*	1 (0.8)	1 (1.0)	0	0.535 ^a^
	*Yes/hospitalization*	7 (5.7)	7 (7.1)	0	
	*Yes/outpatient*	64 (52.0)	50 (51.0)	14 (56.0)	
	*Yes/both*	51 (41.5)	40 (40.8)	11 (44.0)	
Psychiatric medication	*No*	28 (22.8)	27 (27.6)	1 (4.0)	**0.012** ^a^
	*Yes*	95 (77.2)	71 (72.4)	24 (96.0)	

(^a^) *p*-value from chi-square test. Abbreviation: ADHD, Attention Deficit Hyperactivity Disorder; BP, Borderline personality; AP, Antisocial Personality.

**Table 3:** Psychiatric disorders of the AUD group.

Variables *[N (%)]*		AUD N = 122	Male N = 97	Female N = 25	*p*-Value ^a^
Mood disorders	*No*	68 (56.7)	61 (62.9)	8 (32)	**0.003**
	*Primary*	24 (20)	18 (18.6)	7 (28)	
	*Inducted*	18 (15)	14 (14.4)	4 (16)	
	*Both*	9 (7.5)	4 (4.1)	6 (24)	
Psychotic disorders	*No*	112 (92.6)	91 (94.8)	21 (84)	0.158
	*Primary*	3 (2.5)	2 (2.1)	1 (4)	
	*Inducted*	6 (5)	3 (3.1)	3 (12)	
	*Both*	-	-	-	
Anxiety	*No*	94 (77.7)	74 (77.1)	20 (80)	0.404
	*Primary*	18 (14.9)	13 (13.5)	5 (20)	
	*Inducted*	8 (6.6)	8 (8.3)	-	
	*Both*	1 (0.8)	1 (1.0)	-	
Antisocial personality	*No*	116 (95.9)	92 (95.8)	24 (96)	0.970
	*Yes*	5 (4.1)	4 (4.2)	1 (4)	
Borderline personality	*No*	104 (85.9)	85 (88.5)	19 (76)	0.108
	*Yes*	17 (14.1)	11 (11.5)	6 (24)	
ADHD	*No*	100 (82.7)	79 (82.3)	21 (84)	0.841
	*Yes*	21 (17.3)	17 (17.7)	4 (16)	

(^a^) *p*-value from chi-square test. ADHD, Attention Deficit Hyperactivity Disorder.

**Table 4:** Sample description of addiction by DMS-IV and PRISM.

Variables		Alcohol (N = 122)	Male (N = 97)	Female (N = 25)	*p*-Value
Age at first alcohol use *(Mean ± SD)*	*Years*	30.51 ± 11.16	29.28 ± 10.91	35.24 ± 11.05	**0.016** ^a^
Alcohol abstinence *(Mean ± SD)*	*Month*	10.83 ± 18.10	10.50 ± 18.40	11 ± 16.37	0.310 ^a^
Problematic alcohol use *(Mean ± SD)*	*Years*	16.38 ± 11.43	17.34 ± 12.13	12.88 ± 7.14	0.065 ^a^
DSM-IV-TR criteria for AUD *(Mean ± SD)*	*Years*	7.34 ± 2.0	7.56 ± 2.06	6.48 ± 1.33	**0.015** ^a^
Number of abstinence periods (minimum 6 month) *[N (%)]*	0	39 (35.2)	30 (31.6)	9 (36)	0.864 ^b^
	1	49 (40.8)	40 (42.1)	10 (40)	
	2	20 (16.7)	16 (21.1)	4 (16)	
	3	7 (5.8)	6 (7.4)	1 (4.0)	
	4	2 (1.7)	1 (1.1)	1 (4.0)	
	5	2 (1.7)	2 (2.1)	0 (0)	
AUD diagnosis throughout life *[N (%)]*	*No*	-	-	-	0.698 ^b^
	*Abuse*	2 (1.6)	2 (2.1)	0 (0)	
	*Dependency*	7 (5.7)	6 (6.2)	1 (4)	
	*Abuse & Dependency*	113 (92.6)	89 (91.7)	24 (96)	
AUD diagnosis in prior last 12 months *[N (%)]*	*No*	1 (0.8)	1 (1.0)	0 (0)	0.717 ^b^
	*Abuse*	4 (3.3)	4 (4.1)	0 (0)	
	*Dependency*	5 (4.1)	4 (4.1)	1 (4.0)	
	*Abuse & Dependency*	112 (91.8)	88 (90.7)	24 (96.0)	
AUD diagnosis in last year *[N (%)]*	*No*	26 (21.3)	21 (21.6)	5 (20.0)	0.996 ^b^
	*Abuse*	9 (7.4)	7 (7.2)	2 (8.0)	
	*Dependency*	23 (18.9)	18 (18.6)	5 (20.0)	
	*Abuse & Dependency*	64 (52.5)	51 (52.6)	13 (52.0)	
Cocaine *[N (%)]*	*No*	83 (68.0)	61 (62.9)	22 (88.0)	**0.032** ^b^
	*Abuse*	19 (15.6)	19 (19.6)	0 (0)	
	*Dependency*	2 (1.6)	1 (1.0)	1 (4.0)	
	*Abuse & Dependency*	18 (14.8)	16 (16.5)	2 (8.0)	
Cannabis *[N (%)]*	*No*	104 (85.2)	82 (84.5)	22 (88.0)	0.669 ^b^
	*Yes*	18 (14.8)	15 (15.5)	3 (12.0)	
Sedative *[N (%)]*	*No*	114 (93.4)	92 (94.8)	22 (88.0)	0.459 ^b^
	*Yes*	8 (6.5)	5 (5.2)	3 (12.0)	
Stimulants *[N (%)]*	*No*	118 (96.7)	94 (96.9)	24 (96.0)	0.820 ^b^
	*Yes*	4 (3.3)	3 (3.1)	4 (3.3)	

(^a^) *p*-value from Mann–Whitney U test. (^b^) *p*-value from chi-square test. Values in bold are statistically significant when *p* < 0.05.

**Table 5:** Plasma concentrations of inflammation-related factors in abstinent AUD and control subjects.

Variables		Control *Mean ± SD*	AUD *Mean ± SD*	*U*	*p*-Value ^a^
LPS	EU/mL ^b^	0.47 ± 0.44	0.68 ± 0.67	3289	**0.013**
HMGB1	ng/mL	83.96 ± 15.98	107.48 ± 27.41	4987	**˂0.001**
Leptin	ng/mL	5.59 ± 4.71	3.86 ± 3.83	1922	**0.003**
Fractalkine	pg/mL	4.99 ± 3.21	4.21 ± 2.61	129	0.405
MCP-1	pg/mL	43.49 ± 17.99	49.37 ± 20.15	183	0.411
SDF-1α	pg/mL	345.86 ± 163.75	273.75 ± 113.49	131	0.447
IL-1β	pg/mL	2.46 ± 1.00	3.17 ± 1.33	190	0.112
IL-6	pg/mL	3.59 ± 6.90	5.37 ± 6.30	217	**0.012**
IL-10	pg/mL	2.43 ± 5.61	0.93 ± 0.57	131.5	0.707
TNF-α	pg/mL	10.88 ± 4.32	13.21 ± 4.36	192	0.098
GGT	IU/L	14.43 ± 3.99	43.85 ± 40.58	433.5	**˂0.001**
GOT	IU/L	24.00 ± 5.03	29.46 ± 30.47	248.0	0.810
GPT	IU/L	22.43 ± 2.64	39.82 ± 55.80	316.0	0.134

(^a^) *p*-value from Mann–Whitney U test. (^b^) EU, unit of measurement for endotoxin activity. Abbreviations: GGT, gamma-glutamyltransferase; GOT, glutamic oxaloacetic transaminase; GPT, glutamate pyruvate transaminase; HMGB1, high-mobility group box 1 protein, also known as high-mobility group protein 1; IL-1β, interleukin 1 beta; IL-6, interleukin 6; IL-10, interleukin 10; LPS, lipopolysaccharide; MCP-1, monocyte chemoattractant protein-1; SDF-1α, stromal cell-derived factor 1 alpha; TNF-α, tumor necrosis factor alpha.

**Table 6:** Plasma concentrations of inflammation-related factors in abstinent AUD and control male subjects.

Variables		Male Control *Mean ± SD*	Male AUD *Mean ± SD*	*U*	*p*-Value ^a^
LPS	EU/mL ^b^	0.73 ± 0.57	0.73 ± 0.71	596	0.623
HMGB1	ng/mL	84.37 ± 16.28	108.91 ± 23.86	1098	**˂0.001**
Leptin	ng/mL	2.92± 2.19	3.09 ± 2.76	431	0.898
Fractalkine	pg/mL	3.29 ± 1.9	4.08 ± 2.88	37	0.964
MCP-1	pg/mL	35.01± 13.29	50.48 ± 18.03	52	0.151
SDF-1α	pg/mL	281.16 ± 119.55	266.28 ± 96.39	39	0.820
IL-1β	pg/mL	2.29 ± 1.16	3.28 ± 1.11	49	**0.045**
IL-6	pg/mL	1.12 ± 0.96	4.57 ± 3.81	54	**0.007**
IL-10	pg/mL	0.80 ± 0.36	1.01 ± 0.56	38	0.428
TNF-α	pg/mL	9.89 ± 3.00	14.48 ± 3.88	49	**0.045**

(^a^) *p*-value from Mann–Whitney U test. (^b^) EU, unit of measurement for endotoxin activity. Abbreviations: see Table 5.

**Table 7:** Plasma concentrations of inflammation-related factors in abstinent AUD and control female subjects.

Variables		Female Control *Mean ± SD*	Female AUD *Mean ± SD*	*U*	*p*-Value ^a^
LPS	EU/mL ^b^	0.35 ± 0.32	0.54 ± 0.46	567	**0.012**
HMGB1	ng/mL	83.79 ± 16.06	101.69 ± 37.71	577	**0.017**
Leptin	ng/mL	6.51 ± 5.01	6.07 ± 5.42	407.5	0.535
Fractalkine	pg/mL	4.99 ± 3.21	4.21 ± 2.61	23	0.127
MCP-1	pg/mL	51.98 ± 19.05	48.42 ± 22.45	37	0.718
SDF-1α	pg/mL	410.56 ± 186.16	280.16 ± 129.65	26	0.207
IL-1β	pg/mL	2.6 ± 0.93	3.09 ± 1.53	49	0.592
IL-6	pg/mL	5.71 ± 9.12	6.04 ± 7.93	54	0.340
IL-10	pg/mL	3.84 ± 7.61	0.87 ± 0.60	29	0.290
TNF-α	pg/mL	11.72 ± 5.29	12.14 ± 4.61	47	0.711

(^a^) *p*-value from Mann-Whitney U test. (^b^) EU, unit of measurement for endotoxin activity. Abbreviations: see Table 5.

**Table 8:** Diagnostic sensitivity, specificity, and predictive values for selected inflammation-related factors and experimental models for AUD.

Variables	Cutoff Point ^a^	Prognostic Probability (%) ^b^	AUC (%) ^c^	Sensitivity (%)	Specificity (%)
LPS	>0.294 EU/mL	53	62.2	61.2	61.1
HMGB1	>95.94 ng/mL	71	78	71.3	72.1
Leptin	<3.79 ng/mL	55	64.4	68.9	60
SDF-1α	<373.4 pg/mL	35	58	92.3	50
Model 1		78	90.4	86.7	88.9
Model 2		79	86.1	84.1	71.1
Model 1 + GGT ^c^		90	96.7	83.3	93.3
Model 2 + GGT		91	97.4	71.4	94.9
Model for males		72	90.5	100	85.7
Model for females		68	85.9	83.3	84.6

(^a^) Kolmogorov–Smirnov statistics. (^b^) Receiver operating characteristic (ROC) analysis for two discriminative logistic models of pathological use of alcohol whose variables were HMGB1, leptin and SDF-1α for model 1, and HMGB1, leptin and LPS for model 2. Both models were adjusted for sex and age as covariates. The same analysis was performed for the group of male subjects (model male) whose variables were HMGB1 and TNF-α, and for the group of female subjects (model female) with the variables LPS and fractalkine. These models were adjusted for age as covariate. (^c^) AUC, area under the curve; GGT, gamma-glutamyltransferase.

## Data Availability

Data is contained within the article.
